# Supplementary material for: A Spike-like Self-Assembly of Polyaspartamide Integrated with Functionalized Nanoparticles
Source: Polymers (Basel). 2024 Jan 15;16(2):234. doi: 10.3390/polym16020234 (PMC10819371; doi:10.3390/polym16020234)
Supplement: Supplementary file 1 [file polymers-16-00234-s001.zip › polymers-2754031-supplementary.pdf]

Supporting Information

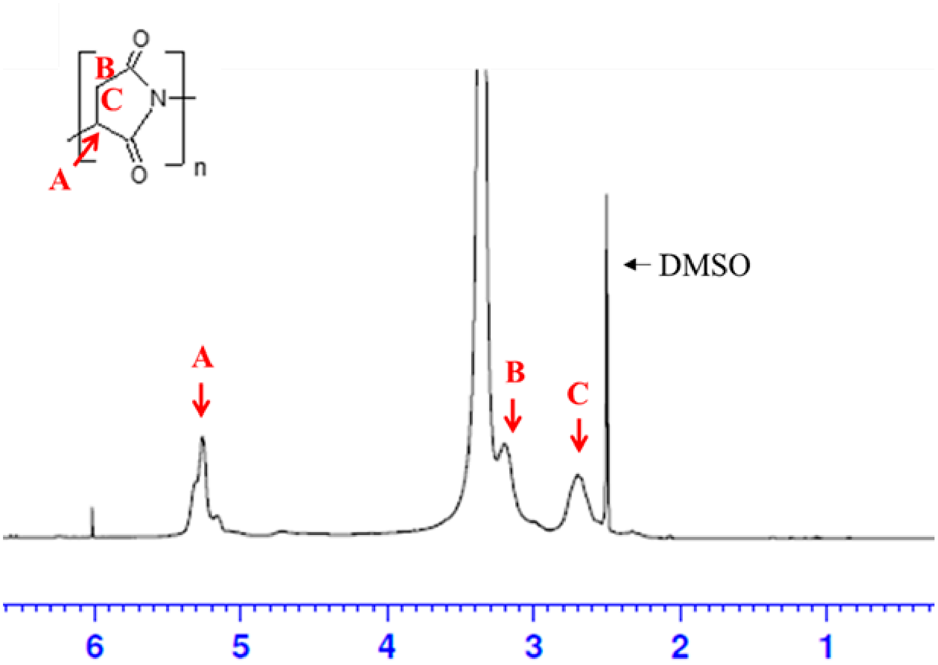

Figure S1. ^{1}H NMR spectra of poly(succinimide) (PSI).

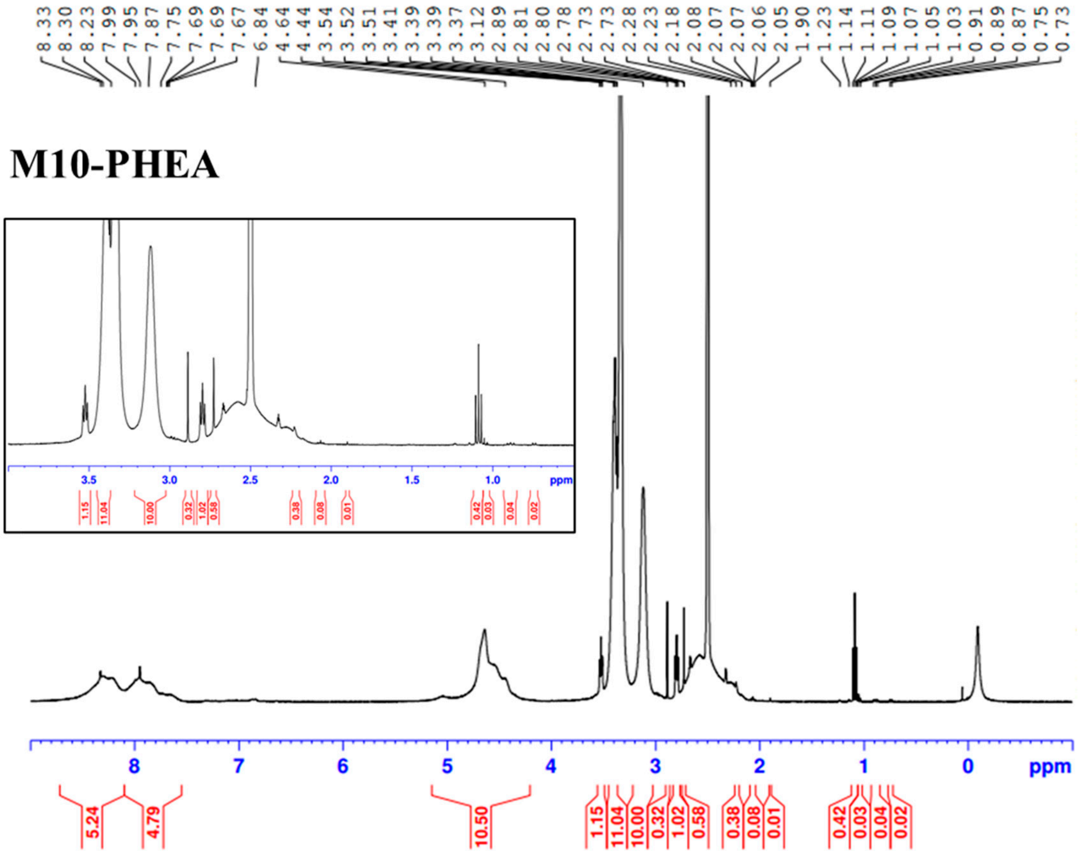

Figure S2. ^{1}H NMR spectra of M10-PHEA (DS$_M$: 10 mol%).

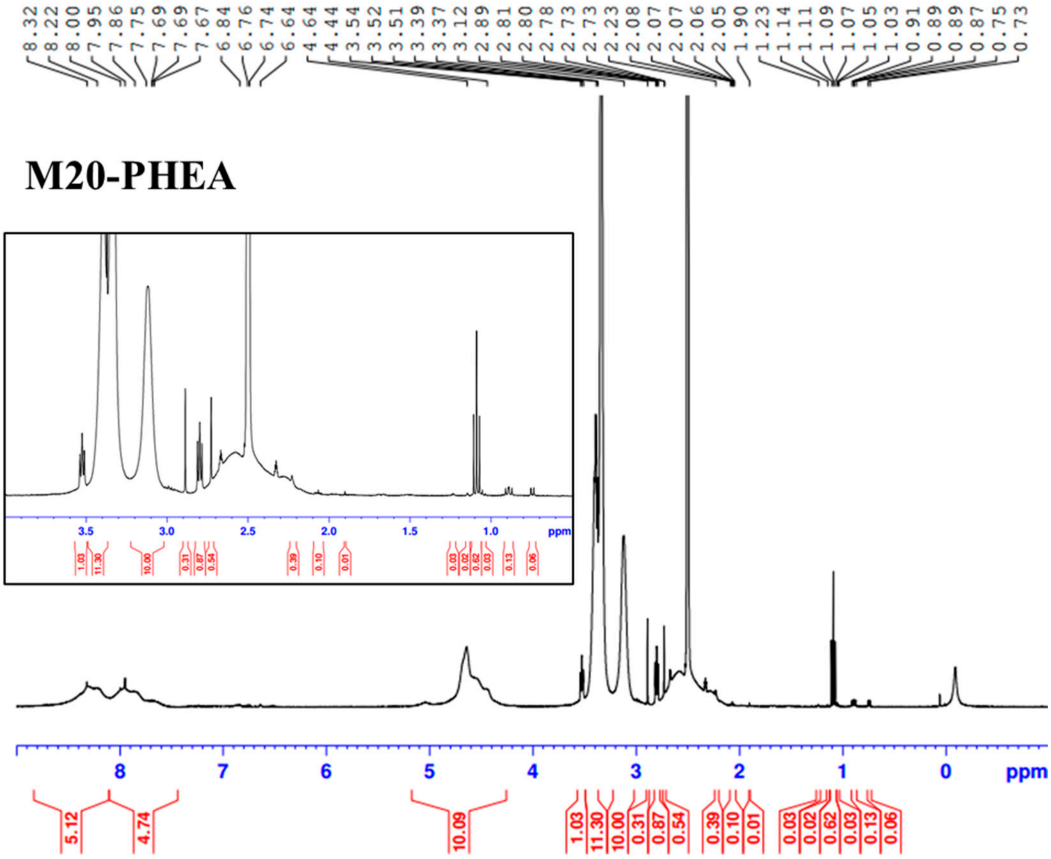

Figure S3. ^{1}H NMR spectra of M20-PHEA (DS$_M$: 20 mol%).

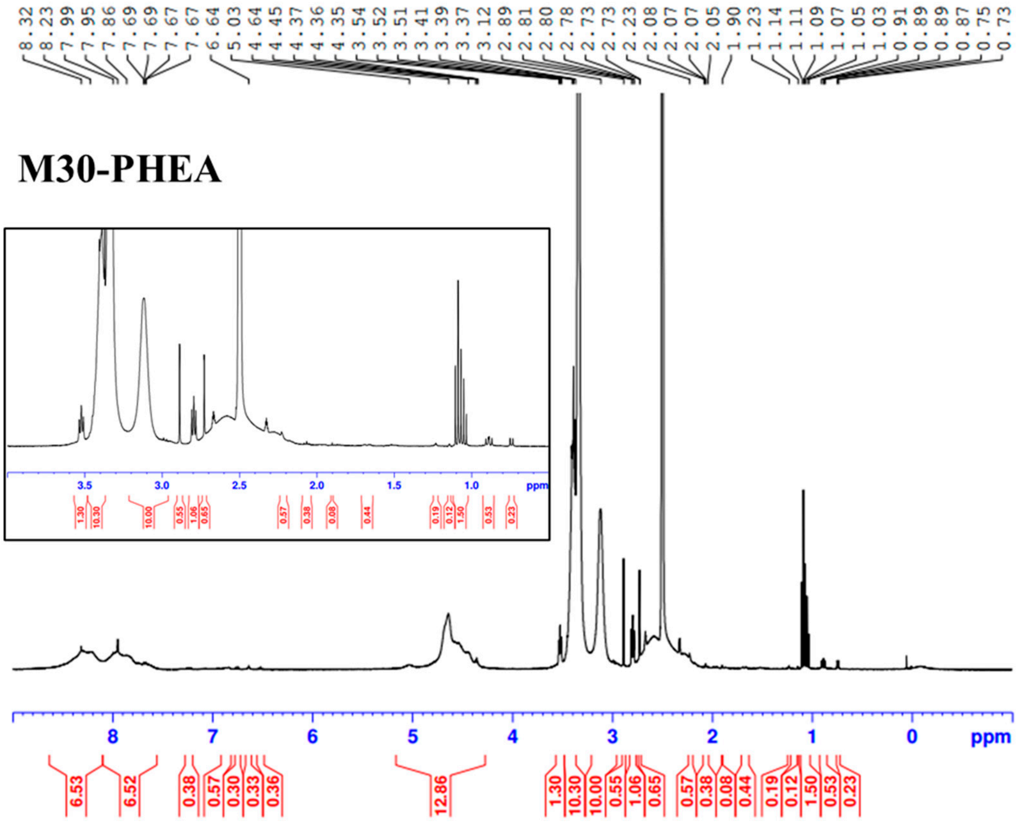

Figure S4. ^{1}H NMR spectra of M30-PHEA (DS_M: 30 mol%).

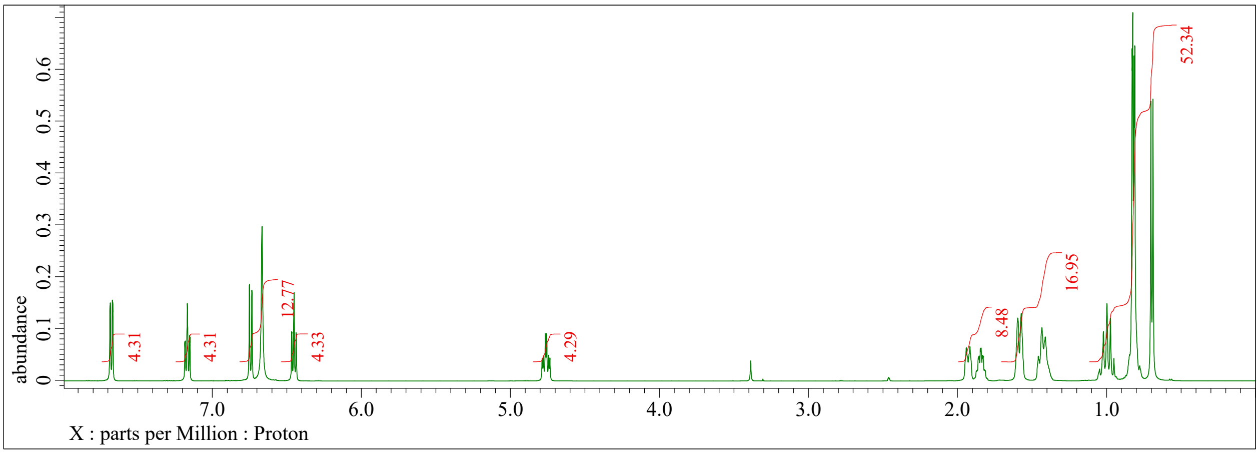

Figure S5. ^{1}H NMR spectra of Mentyl Antharnilate.

Table S1. Ingredients of Oil-in-Water Emulsion Used for UV-Protection Test

phase	INCI name	phase	INCI name
A	DIwater	C	cetearyl olivate, sorbitan olivate
	ethylene diamine tetraacetic acid		PEG-100 stearate, glyceryl stearate
	betaine		cetearyl alcohol
	glycerin		glyceryl monostearate
	sodium hyaluronate		stearic acid
	glycerine, glyceryl acrylate, acrylic acid copolymer, water		sorbitan monostearate
B	xanthan gum		dicaprylyl carbonate
	carbomer		pentaerythrityl tetraethylhexanoate
D	DI water		dimethicone
	tromethamine		vitamin E acetate
E	hydroxyethyl acrylate, sodium acryloyl-dimethyl taurate copolymer, polyisobutene,PEG-7 trimethylolpropane coconut ether		Cyclopentasiloxane
F	caprylyl glycol, ethylhexyl glycerin	G	TiO_2@C_{18}-M-PHEA

The sunscreen emulsions were formulated using an oil-in-water emulsion system. Initially, phase A, consisting of a sequestering agent and moisturizer, was homogenized by heating to 80°C for uniform mixing. Subsequently, phases B and C, comprising a viscosity-increasing agent and surfactant within the oil phase, were individually heated and mixed. These B and C phases were sequentially incorporated into phase A. The combined A, B, and C mixture underwent homogenization at 3000 rpm for 5 minutes while maintaining the temperature at 80°C. Further, phases D, E, F and G were introduced sequentially

during a secondary mixing step at 3000 rpm for an additional 5 minutes. Following emulsification, the mixture was gradually cooled to room temperature.

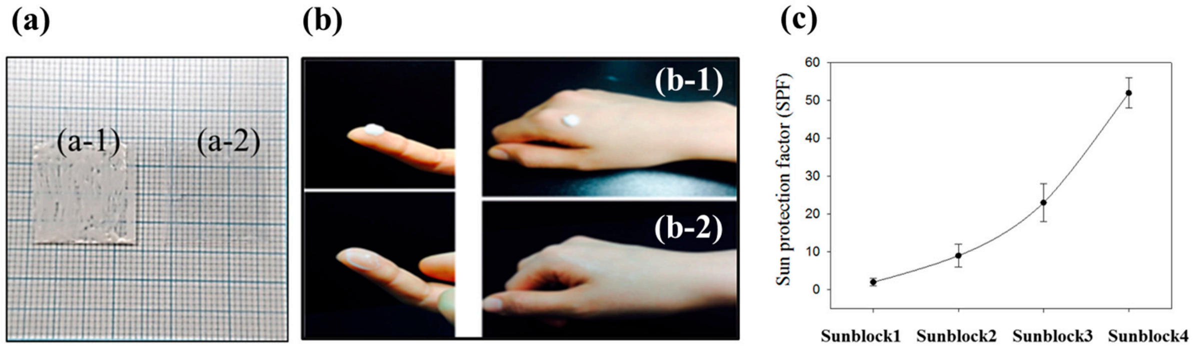

Figure S6. Comparison of transparency between (a-1, b-1) an O/W emulsion cream formulated with TiO_2 NPs and (a-2, b-2) an O/W emulsion cream formulated with TiO2@C_{18}-M-PHEA, applied to the film (a) and the skin (b) at a concentration of 3.6 mg/cm^2. In (c), the O/W emulsion cream formulated with TiO_2@C_{18}-M-PHEA, denoted as Sunblock 4, exhibited an SPF index of approximately 50. Compared to formulations using only TiO_2 NPs at higher concentrations (Sunblock 1, 2, 3), Sunblock 4 demonstrated a reduction in opaqueness while showing an increase in UV-blocking efficacy.
